# Overlooked sex and gender aspects of emerging infectious disease outbreaks: Lessons learned from COVID-19 to move towards health equity in pandemic response

**DOI:** 10.3389/fgwh.2023.1141064

**Published:** 2023-02-20

**Authors:** Lynn Lieberman Lawry, Roberta Lugo-Robles, Vicki McIver

**Affiliations:** ^1^ Department of Preventive Medicine and Biostatistics, Uniformed Services University, Bethesda, MD, United States; ^2^ Department of Preventive Medicine and Biostatistics, Henry M. Jackson Foundation, Bethesda, MD, United States; ^3^ Clinical Pharmacist Belle Center, OH, USA

**Keywords:** sex, gender, emerging infections diseases, COVID-19, pandemics, epidemics, health equity

## Abstract

Sex and gender issues are especially important in emerging infectious diseases (EIDs) but are routinely overlooked despite data and practice. Each of these have an effect either directly, *via* the effects on vulnerability to infectious diseases, exposures to infectious pathogens, and responses to illness, and indirectly through effects on disease prevention and control programs. The severe acute respiratory syndrome coronavirus-2 (SARS-CoV-2), the viral agent of coronavirus disease 2019 (COVID-19) has underscored the importance of understanding the sex and gender impacts on pandemics. This review takes a broader looks at how sex and gender impact vulnerability, exposure risk, and treatment and response that affect incidence, duration, severity, morbidity, mortality, and disability of EIDs. And although EID epidemic and pandemic plans need to be “pro-women”, they need to be broader and include all sex and gender factors. Incorporation of these factors are a priority at the local, national, and global policy levels to fulfil the gaps in scientific research, public health intervention programs and pharmaceutical service strengthening to reduce emerging disease inequities in the population during pandemics and epidemics. A failure to do so creates acceptance of the inequities and infringes on fairness and human rights norms.

## 1. Introduction

Gender, usually limited to men and women, has focused on how roles, norms and behaviors impact outcomes related to disease prevention and control programs, whereas, sex differences have focused on pregnancy, anatomical and immunological differences and have been more female or women focused (1–4). The full spectrum includes sex, defined as male, female or intersex based on sex chromosome complement, reproductive tissues and sex steroid hormone and sexual and gender minorities (lesbian, gay, bisexual, transgender, and queer/questioning) impacts, not just cis- or binary gender impacts, on pathogenesis, health outcomes, mortality, and morbidity in EIDS (5).

**Figure 1:**
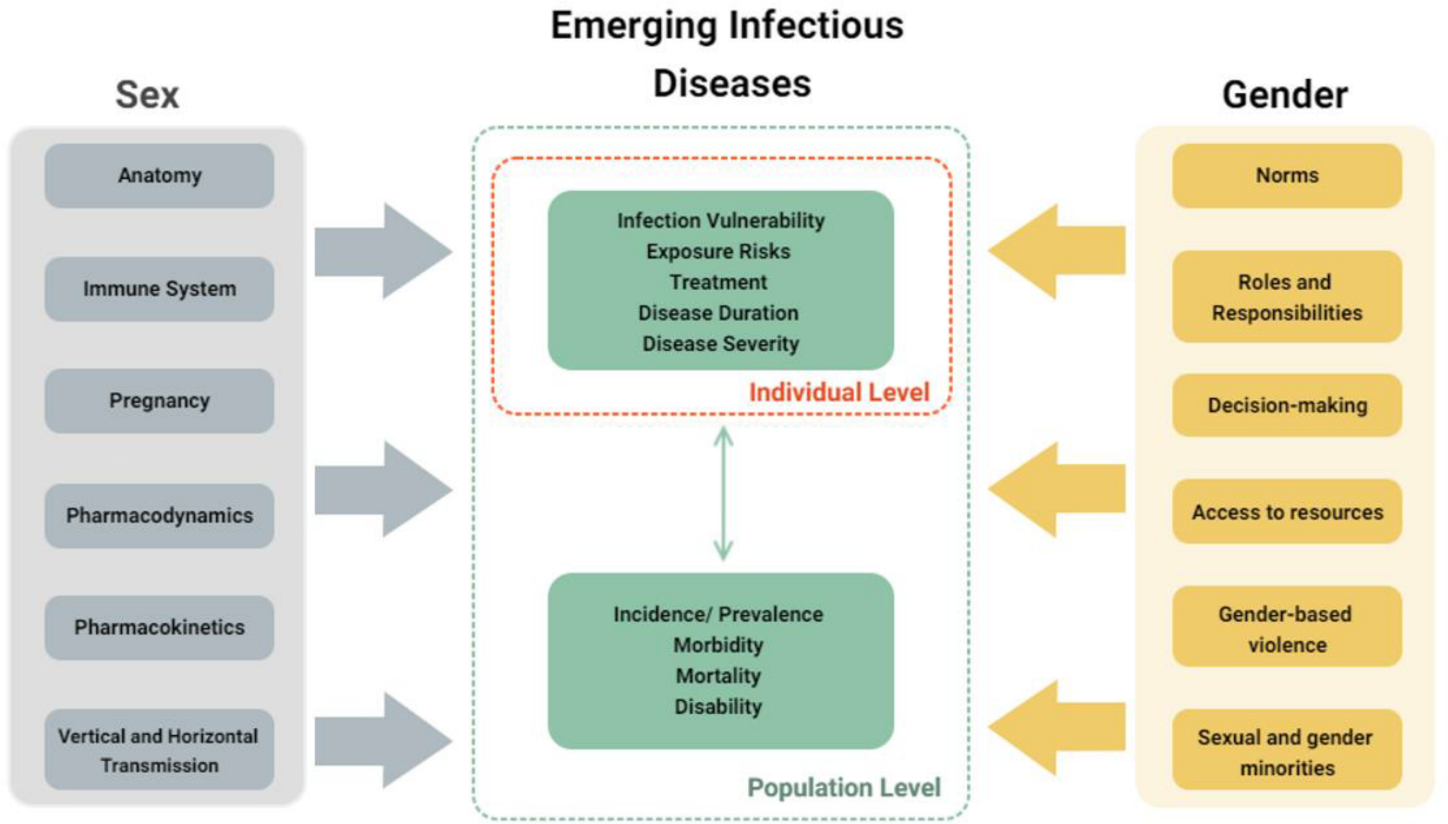
Conceptual framework for sex and gender effects in emerging infectious diseases.

Sex and gender factors have an effect either directly, *via* the effects on vulnerability to infectious diseases, exposures to infectious pathogens, and responses to illness, as well as indirectly through effects on disease prevention and control programs (2, 3) (Figure 1).

Emerging infectious diseases are infectious diseases that have newly appeared in a population and include pandemics and epidemics like COVID-19, Ebola, Zika, Middle East respiratory syndrome (MERS) and severe acute respiratory syndrome (SARS) (3). The COVID-19 pandemic and other EIDs have shown that sex and gender matter in risk, mortality, and morbidity (2, 6–11). This review will take a broader look at the sex- and gender-specific aspects of epidemics and pandemics, using lessons learned from COVID-19, to better define and outline the pathways by which a person's sex and gender affect outcomes from EID outbreaks and how to reduce inequities during EID pandemics and epidemics.

### 1.2. Sex related factors

Physiological and biological factors define males and females and include chromosomal, hormonal, and anatomical characteristics (5, 12). In general, EID frameworks focus on sex related factors of anatomy, immune system, and pregnancy, leaving out other important factors such as pharmacokinetics, pharmacodynamics and vertical and horizonal transmission; all important issues that affect outcomes from EIDs (2, 3).

### 1.3. Physiological sex related factors

Genes located on the X chromosome can be expressed at a higher level in females than in males due to two X chromosomes (13). For example, specific encoding for genes on the X chromosome are involved in the regulation of immunity that are differently represented in males and females (13, 14).

#### 1.3.1. Sex steroid hormones and immunity

Sex steroid hormones can alter gene expression to have a protective role and may account for female's ability to mount a more vigorous immune response to infections, produce greater antibody responses to vaccines, and may explain lower fatality rates among females (15–17). Estrogen and testosterone levels change with age and therefore age and sex-disaggregated data is also important to determine immune status throughout life-cycles as the ability to mount an immune response changes over time with exogenous supplement of hormone therapy such as in post-menopausal persons, gender transition or those with reproductive cancers (17–19).

#### 1.3.2. Sex and mortality

The possible differences in expression of ACE2 on the X chromosome between males and females; may partially explain the sex disparity in morbidity and mortality from COVID-19, and especially the lower mortality rates among females (20–22). During MERS, the higher mortality among males was thought to be due to different expression of DPP-4 however, these differences were not evident and the higher mortality appears to be gender related and not sex related since DPP-4 levels are higher among smokers and those with chronic obstructive pulmonary disease which are more commonly associated with males (23, 24).

#### 1.3.3. Pharmacokinetics and pharmacodynamics

Males, females and pregnant vs. non-pregnant individuals have sex related factors that affect drug absorption, distribution, metabolism, and elimination (2, 3, 25). Factors that influence drug absorption are route-specific (oral, dermal, rectal, etc.) and may also be sex-specific (25). As such, females have more adverse drug events (ADE) including overdoses and antimicrobial resistance (AMR) than males (25). Each of these pharmacokinetic factors play a major role in treatment outcomes, ADE and potentially antimicrobial resistance in EIDs (20, 22, 24–26).

There are higher mortality rates in post-menopausal women than reproductive age women in COVID-19, it is therefore important for healthcare providers to understand the pharmacokinetic changes due to hormonal status (pregnancy, OCPs, menopause, hormone replacement or deprivation therapy) to avoid over- or underdosing female patients during treatment for COVID-19 (20–22, 27).

The pharmacokinetic sex differences in the phases and enzymes that control drug metabolism differ between ethnicity, males and females and are compounded by pregnancy and and the use of oral contraceptives (27). Females have less clearance and a longer half-life of some drugs such as benzodiazepines and antibody treatments (e.g., Remdesivir), which are used for sedation if a ventilator is needed (25). Clinical trials investigating pharmacological therapies for COVID-19, have lacked sex-disaggregated data (28). Drug pharmacokinetics are significantly altered during pregnancy due to changes in drug distribution, absorption, metabolism, and excretion making some drug sub-therapeutic during pregnancy (25, 29).

Hormonal changes and hormonal replacement therapy can also lead to altered drug disposition in females. Due to the differences in drug metabolism, for example, females require 22.0% less of a dose of vecuronium than men, a neuromuscular blocker used for anesthesia (30). Vecuronium is itemized on the United States Food Drug Administration (FDA) list of COVID-19 drugs in short supply which among other sedatives (31). Given these shortages, it is vital to understand the pharmacokinetic and pharmacodynamic sex differences to avoid ADE, and to tailor supply chain management and rational use to care for patients requiring mechanical ventilation.

Investigational drugs used off label in COVID-19 patients play a role in sex related pharmacokinetics among the autoimmune disease population; 78.0% of whom are female. Hoarding of hydroxychloroquine, without scientific evidence to support its use, as a treatment in COVID-19, disproportionately affected females (32).

#### 1.3.4. Pharmacodynamics and vaccines

Females have higher innate immunity, humoral and cell-mediated immune responses or adaptive immunity to antigenic stimulation, vaccination and higher titers to vaccination, and infection than males (15, 33). Females consistently report more adverse reactions than males in response to vaccines including COVID-19 (15, 34). However, in those who are 65 years and older, antibody responses are lower in both males and females compared with younger adults which is why there is a high-dose influenza vaccine (15, 33). Females also have better efficacy (the percent reduction in disease incidence in a vaccinated population) which means lower rates of hospitalization and mortality in older females when compared to males (15). It is therefore, important for COVID-19 vaccine and any other vaccine developed to prevent the consequences of an EID adverse response reporting to include sex- and age-disaggregated data and include hormonal status of vaccinated individuals to ensure efficacy, mortality and/or morbidity are understood given the sex-based pharmacodynamics of vaccine induced immunity.

### 1.4. Biological sex related factors

#### 1.4.1. Pregnancy

Pregnant individuals are vulnerable to some infectious diseases including nosocomial outbreaks such as influenza A (H1N1), Ebola, and Zika (35). Unlike with MERS and SARS, where the mortality rate for pregnant individuals was 25.0%–35.0%, the mortality rate is 0.9% for COVID-19 (35). However, pregnant women with COVID-19 are more likely to need admission to an intensive care unit and need ventilation and are at increased risk for preterm birth, preeclampsia and caesarean and perinatal death (35, 36). The reproductive health needs of pregnant individuals, during epidemics and pandemics, due to reductions in access to family planning services, increasing the risk of unplanned pregnancies and pregnancy care (37). During the West Africa Ebola outbreak, hospital and health clinic assisted births dropped by 30.0% and maternal mortality increased 75.0% (38). Indirect deaths of maternal and neonatal and stillbirths caused by the epidemic outnumbered direct Ebola-related deaths (39). This issue is not just in low and middle-income (LMIC) countries. COVID-19 revealed that even in industrialized countries, partly due to lockdowns, there was reduced care seeking and hospital births (37, 40–42). And the move to newer models of care, such as telehealth disproportionately affected care for those who did not have smart phones, computers, and/or access to the internet (43).

#### 1.4.2. Male fertility

Viruses such as Zika, Ebola, SARS, SARS-CoV-1 and Marburg, can cause viral orchitis, which have the potential to affect spermatogenesis and testosterone production (44). Patients with moderate COVID-19 infection have significantly lower sperm concentration compared to patients with mild infection (44). There are currently no longitudinal studies on fertility outcomes of those infected with SARS-CoV-2, however, given the sperm and hormonal changes, as well as potential testicular damage and subsequent infertility mediated through viral invasion or secondarily *via* immunological or inflammatory response, especially among young adults and pediatric patients, further investigation will be necessary (44).

#### 1.4.3. Vertical transmission

Vertical transmission is defined as the passage of an infectious pathogen from the mother to the fetus during the antepartum and intrapartum periods, or to the neonate during the postpartum period *via* the placenta *in utero*, body fluid contact during childbirth, or through direct contact owing to breastfeeding after birth (45). There are reports of vertical transmission of SARS-CoV-2 (approximately 3.2%) thus far, largely in the third trimester (45). However, there is not enough data to determine the overall rates of vertical transmission in early pregnancy nor the potential risk for consequent fetal morbidity and mortality (45). There is significant concern about vertical transmission given the presence of the ACE2 receptor in the placenta and that SARS and MERS have been associated with severe maternal and neonatal morbidity and mortality and adverse pregnancy outcomes (miscarriage, preterm birth, and stillbirth) (46). Yet, there were no known cases of vertical transmission during SARS and MERS despite the associated poor outcomes of pregnancy and the infections and little evidence at the current time of the presence of SARS-CoV-2 in fetal tissues (45, 46). The concern of vertical transmission is due to the known teratogenicity and fetal morbidity associated with other viral infections such as Zika (45). There are insufficient data to conclude vertical transmission through breastfeeding in COVID-19, as in Ebola, SARS and Zika; where the virus was detected in breast milk of survivors but transmission to infants is unknown (47–51). Without evidence-based research, discouraging breastfeeding can have significant effects on infant mortality and morbidity and setback public health efforts and gains in promoting breastfeeding worldwide (52, 53).

#### 1.4.4. Horizontal transmission

Among Ebola male survivors, the virus remains in seminal fluid and other body fluids of all survivors for prolonged periods of time (54). And there is evidence that Ebola, latent in seminal fluid, is sexually transmitted and consequently poses a risk to willing and unwilling partners. Female reproductive organs do not express ACE2 like male gonads, therefore, horizontal transmission (sexual transmission) of SARS-CoV-2 infection is a possibility but given the low number of studies that detected viral RNA in semen, further study is necessary (44, 55, 56). Evidence of the impact of SARS-CoV-2 infection on male reproduction, as well as the potential of SARS-CoV-2 viral transmission through seminal fluids, remains inconclusive (44, 55, 56).

### 1.5. Gender related factors and their effect on emerging infectious diseases

Gender is defined as the socially constructed roles, behaviors, activities, and attributes that a given society considers appropriate for males and females (5). Gender often plays an important role in the capacity and willingness of individuals, households, and communities to protect themselves from infection and obtain treatment when they become ill (2, 3). Gender norms, roles and responsibilities, decision-making and access to resources impact incidence and severity, morbidity, mortality and disability in EIDs but have been women-focused (2, 3). COVID-19 reduced access to gender-affirming resources and the ability of transgender and non-binary people to live according to their gender globally, increased the rates of mental health disorders, and increased social isolation and violence among transgender and non-binary individuals (57). Pandemics exacerbate existing gender inequalities, thus, gender needs to be broadened to cover males, females, intersex, cisgender, transgender and non-binary persons and gender analyses sub-grouped by different gender groups.

#### 1.5.1. Gender norms

Gender-related norms and behaviors can increase risk factors and vulnerability to infectious diseases such as smoking which may be a risk factor for increased fatality among men who have COVID-19 (10, 26, 58). Gender-based violence (GBV) is rooted in gender norms which defines the accepted social expectations of typical and appropriate behaviors among genders (59). Where expectations and behaviors change during emergencies, they end in increases in violence and poor health outcomes (39, 60).

#### 1.5.2. Roles and responsibilities

Women make up 70.0% of the healthcare and social services workforce putting them on the frontlines, sometimes without adequate personal protective equipment, of epidemics and pandemics while also serving as caregivers for those who become sick at home (61, 62). During the Ebola epidemics women were exposed due to their occupational, traditional and care giving roles (63).

In addition to the sex related hormonal and immunologic differences between males and females, it is possible the excess in male mortality might be due to gender differences in health-seeking behavior (with women reporting they visited their primary care provider to a greater extent) (64). However, pre-existing lack of resilience in health systems in addition to the lack of trust of healthcare providers or services at the time of the epidemic, has been reported to contribute to reduced utilization of healthcare services seen in Ebola outbreaks, especially for maternal health care and for other health issues such as human immunodeficiency virus (HIV)/acquired immunodeficiency syndrome (AIDS), tuberculosis, and malaria (36–39, 63, 65).

#### 1.5.3. Decision making and access to resources

A full spectrum of reproductive health services are generally an afterthought during emergencies such as a pandemic or, as we have seen in COVID-19, become politicized and purposely limited (66, 67). In doing so, any limits to reproductive health care exacerbate health inequities and social injustices and lead to an increase in maternal and child mortality (67, 68). Quarantine measures and higher morbidity from COVID-19 have put increased strain on women financially compared to men (69). The ability for women to sustain livelihoods has become more difficult due to increased childcare responsibilities and higher participation in the informal sector and casual work that has been limited by COVID-19 ultimately putting them at risk for violence (60, 62, 63, 69).

#### 1.5.4. Gender-based violence (GBV)

It is well known that GBV increases following EIDs outbreaks (70, 71). The COVID-19 pandemic triggered extensive and severe economic and social stresses. When combined with pre-existing toxic social norms and gender inequalities, strict quarantine measures including “stay at home” orders and social distancing, and the disruption of access to support services, GBV, domestic homicides and child abuse have all increased just as in other EID epidemics such as Ebola and HIV (60, 67, 70, 71).

#### 1.5.5. Sexual and gender minorities

Sexual and gender minority (SGM) populations are an underserved and marginalized population that has been affected disproportionately by the social, psychological, financial, physiological, and mental health impacts of COVID-19 (57). Compared with cisgender or heterosexual groups, SGM populations are more immunosuppressed due to HIV, cancer, hormonal treatment, tobacco use and higher rates of comorbid conditions that are known risks for mortality from EIDs and are less likely to seek health care due to stigma, discrimination and social inequities (57, 71). For SGMs, the pandemic exacerbated the lack of social supports due to social distancing and stay-at-home orders resulting increasing the mental health burden and increases in gender based violence (57, 72–74). Gender affirming care was delayed due to postponement of non-emergency care/surgery and restrictions on travel for surgeries (74). Time sensitive puberty suppression, especially for adolescents, was more difficult to access in addition to access to courts to obtain legal documentation for name and gender marker changes required for employment (72–74). A lack of cultural awareness and responsiveness among healthcare practitioners and the overall lack of adequate surveillance data has resulted in unnecessary healthcare delays exacerbated by the effect of the pandemic on SGMs (57, 72–74).

### 1.6. Preventing sex and gender inequities in pandemic planning

#### 1.6.1. Understanding the full scope of sex and gender implications in EIDs

Sex, gender and EID interactions should not be compartmentalized. An inclusive gender assessment that covers sex and all genders is necessary at baseline, early recovery, and post-disaster phases to better understand how EID policies, programs and interventions respond to or will hinder the different needs of the population. Data from a gender analysis gives countries the ability to advocate and raise awareness through social behavior change messaging of harmful traditional and cultural practices that leave individuals and groups vulnerable.

#### 1.6.2. Balancing public health measures and safety

Governments and policymakers cannot forget the role gender plays in GBV including violence against SGMs. Response to violence must be considered an essential service in pandemic preparedness. Disaster preparedness needs to include initiatives that promote sex and gender equity that identify and meet the needs of minority populations, ensuring there is vaccine equity and vaccine strategies are gender-responsive (71–75). The time to think practically about engaging women and other vulnerable groups is before vaccines arrive and not afterwards when the logistics of vaccination will preclude equitable distribution and outreach to marginalized populations (75).

Population level measures such as lockdowns; borders closures; school closures and quarantine measures are powerful tools but also lead to violence risk (71). Consequently, governments and public health agencies when imposing movement restrictions and quarantine measures during an emergency must evaluate and consider cultural norms and population characteristics to minimize the risks of exploitation and abuse.

#### 1.6.3. Limitations in data collection for action

The limitations of data collection systems and reporting infrastructure to disaggregate, report and share data by sex, age and ethnicity and have largely imposed a barrier to analyze sex differences of COVID-19 patients (72, 73). Furthermore, comprehensive sex and sexual orientation and gender identity characteristics have not been included in morbidity and mortality reporting largely due to an inability of data collection systems to identify anything other than cisgender (72). Sex-, gender-, hormone- and ethnicity-disaggregated data are essential for understanding the distributions of risk, infection, and disease in the population, and the extent to which sex and gender play a role in risk, treatment and clinical outcomes.

#### 1.6.4. Understanding the role of sex and gender in pharmacokinetics and pharmacodynamics for supply chain management

The COVID-19 pandemic has shown a need to institute a robust forecasting and supply chain management of drugs to eliminate stock-outs and shortages and a careful analysis of ADE given its sex-related potential and AMR due to potentially higher rates of STIs from increased rates of GBV (76). To avoid preventable maternal and newborn deaths, reproductive health services must be considered essential services (66). Finally, as vaccines are implemented, adverse response reporting must include sex-, age- and hormonal disaggregated data to ensure efficacy, and reduce mortality and/or morbidity.

## 2. Conclusion

Despite decades of understanding that sex and gender impact health, public health and disease, these impacts are routinely overlooked during pandemics (77, 78). Perhaps these concepts are forgotten due to the urgency of new diseases (2, 3, 8, 65). Data and practice from years of experience during epidemics have shown our practices need to be broader and include all sex and gender factors that are a risk for morbidity and mortality. Health equity means we do not avoid inequalities, but we understand, analyze, and overcome them to create fairness and respect for human rights norms by striving for the highest possible standard of health for all people and giving special attention to the needs of those at greatest risk of poor health (78).

## Author contributions

LLL: Conceptualization. LLL, RLR, VM: Writing—original draft preparation. RLR: Visualization. LLL: Supervision. LLL, RLR, VM: Writing—reviewing and editing. All authors contributed to the article and approved the submitted version.

## Conflict of interest

The authors declare that the research was conducted in the absence of any commercial or financial relationships that could be construed as a potential conflict of interest.
